# Physicians' social competence in the provision of care to persons living in poverty: research protocol

**DOI:** 10.1186/1472-6963-10-79

**Published:** 2010-03-25

**Authors:** Christine Loignon, Jeannie L Haggerty, Martin Fortin, Christophe P Bedos, Dawn Allen, David Barbeau

**Affiliations:** 1 Université de Sherbrooke, Faculty of Medicine, Department of Family Medicine, Québec, Canada; 2 McGill University, Department of Family Medicine, Québec, Canada; 3 McGill University, Faculty of Dentistry, Québec, Canada; 4 McGill University, Programs in Whole Person Care, Québec, Canada; 5 Université de Montréal, Faculty of Medicine, Department of Family Medicine, Québec, Canada

## Abstract

**Background:**

The quality of the physician-patient therapeutic relationship is a key factor in the effectiveness of care. Unfortunately, physicians and people living in poverty inhabit very different social milieux, and this great social distance hinders the development of a therapeutic alliance. Social competence is a process based on knowledge, skills and attitudes that support effective interaction between the physician and patient despite the intervening social distance. It enables physicians to better understand their patients' living conditions and to adapt care to patients' needs and abilities.

**Methods/Design:**

This qualitative research is based on a comprehensive design using in-depth semi-structured interviews with 25 general practitioners working with low-income patients in Montreal's metropolitan area (Québec, Canada). Physicians will be recruited based on two criteria: they provide care to low-income patients with at least one chronic illness, and are identified by their peers as having expertise in providing care to a poor population. For this recruitment, we will draw upon contacts we have made in another research study (Loignon et al., 2009) involving clinics located in poor neighbourhoods. That study will include in-clinic observations and interviews with physicians, both of which will help us identify physicians who have developed skills for treating low-income patients. We will also use the snowball sampling technique, asking participants to refer us to other physicians who meet our inclusion criteria. The semi-structured interviews, of 60 to 90 minutes each, will be recorded and transcribed. Our techniques for ensuring internal validity will include data analysis of transcribed interviews, indexation and reduction of data with software qualitative analysis, and development and validation of interpretations.

**Discussion:**

This research project will allow us to identify the dimensions of the social competence process that helps physicians establish therapeutic relationships with low-income patients living with chronic illness. This study will also offer concrete recommendations for improving health interventions among low-income patients and for helping them to better manage their chronic illnesses. Ultimately, our aim is to strengthen the capacity of the health care system and of professionals to provide care that is adapted to the social conditions of people living in poverty.

## Background

Living in conditions of poverty increases the risk of developing chronic illness [1,2]. People living in poverty are at greater risk of experiencing deteriorating health, of developing chronic illnesses and, consequently, of dying prematurely [3-5]. Added to this is the fact that poor people are the least well-served in terms of health care services (*inverse care law*)[6]: they are among those least likely to have a family physician [7], and they more often report having had unmet health needs, compared with people who are more well-off [8]. Finally, they often have negative health care experiences and sometimes feel judged by the physicians who treat them [8-12].

This last point is very important, because the quality of the therapeutic relationship between physicians and patients is a key factor in the effectiveness of care [10,13,14]. In particular, a good therapeutic relationship contributes to patients' empowerment for the self-management of chronic illness. Unfortunately, physicians and people living in poverty inhabit very different social milieux, and this social distance hinders the development of a therapeutic alliance. On one hand, people living in poverty find it difficult to understand the language used by physicians, as well as their recommendations, which are often expressed in biomedical terminology [15,16]. Conversely, physicians have a hard time understanding poor people and are frustrated when these patients do not follow their recommendations. This results in a negative attitude among physicians [17-20], who tend to be more directive with these patients, spend less time with them, and give them less information on treatments[17] Physicians also feel overwhelmed and ill-equipped in these consultations because these patients accumulate many health problems [21-24].

In brief, it is difficult for physicians to establish good therapeutic relationships with their patients when there is a significant social divide between them. To overcome this social divide, physicians need "social competence". Social competence is a process based on knowledge, skills and attitudes that support effective interaction between the physician and the patient despite the intervening social distance. It enables physicians to better understand their patients' living conditions and to adapt care to patients' needs and abilities.

### Physicians dealing with people living in poverty

The literature reports that physician behaviours vary according to patients' socio-economic status [24]. Studies on physicians' experiences with low-income patients have generally concentrated on the influence on medical practice of patients' individual characteristics (e.g. attitudes, language, etc.) [25]. In addition, physicians spend less time with low-income patients, give them less information, and put less effort into encouraging their compliance [17]. They also experience frustration, which leads them to develop a negative attitude toward people living in poverty [21].

Health professionals, particularly physicians, have very little understanding of their low-income patients' social situation. This lack of knowledge about poverty, as well as the mistaken perceptions of poverty held by health professionals, affects the quality of clinical interactions [25]. According to a study conducted among residents in medicine, 25% thought that poverty was a consequence of laziness, 50% thought that the poor were more likely to abuse the health care system, and 50% thought that the poor were less attentive to their health than the rest of the population [26]. Health care providers, who are close to patients' personal and day-to-day experiences, occupy an important position that has a major impact on people's lives [27].

### People living in poverty, dealing with physicians

According to a recent study, people who are vulnerable in terms of health and people who see themselves as poor tend to report less satisfactory health care experiences and more unmet needs [28]. People with low incomes are particularly sensitive to physicians' attitudes and to what physicians say in medical consultations. They are more receptive to advice coming from their physician than from any other source (other professionals, media, brochures, etc.) [24,29].

Recent studies report that poor patients feel stigmatized because of their social status and perceive a lack of sensitivity among health professionals regarding their living conditions [10,14,30]. One of these studies, conducted among low-income Canadians, emphasized that the lack of empathy, compassion and respect they experienced from health care providers had a negative impact on their utilization of services [30]. A recently published book of biographical accounts of women living in poverty reveals that these women, in addition to experiencing deterioration in their health, encountered a lack of understanding, of sensitivity, and of respect among some of their physicians [31]. These women considered that inadequate health care (not having access to a physician's care and feeling judged during health care encounters) had exacerbated their health status. These data confirm the importance of providing physicians with the tools they need to adapt better to the needs of patients living in poverty.

### Limitations of studies and of conceptual approaches

The majority of studies that have looked at relationships between physicians and patients present certain limitations. First, in terms of methodology, they are most often based on quantitative approaches [30,32,33]. Using questionnaires can limit the ability to explore in depth certain avenues that can, however, be explored in detail by using qualitative interviews [22]. In terms of theoretical perspective, few studies have looked at health care relationships as an *interpersonal class process *[34]. Thus, according to Allman, theoretical models, and the studies based on them, do not allow for consideration of the whole picture within which health care is provided [35]. Finally, to our knowledge, while there exist models and studies on cultural competence, to date there is no conceptual model on social competence.

### Theoretical approach and conceptual model for social competence

One of this study's premises is to consider the physician-patient relationship as a tracer for the *interpersonal experience of social class*. Thus, physicians who look after people living in poverty encounter a social distance because they occupy a privileged and prestigious position in our society. As mentioned above, the literature indicates that this difference in social class creates relational difficulties not only for the physician but also for the patient. Our central research premise holds that these difficulties can be overcome by adequate care that requires a social competence process among physicians.

There is currently no model of the physician-patient relationship in a context of social differences, such as, for example, between physicians and people living on unemployment benefits, where the social distance is very great [22,36]. Moreover, the growth in the phenomenon of cultural diversity in the United States has given rise to a proliferation of studies that have produced models of care in an intercultural context. Examples include Bennett's (1986) model of the development of intercultural sensitivity and, more recently, Campinha-Bacote's (2002) cultural competency model [37,38]. Bennett's model has six levels (denial, defense, minimization, acceptance, adaptation, integration) for categorizing the physician's position according to his or her openness to taking cultural differences into account when treating a patient. Campinha-Bacote's model deals with cultural competence and has five dimensions: 1) cultural awareness; 2) cultural knowledge; 3) cultural skill; 4) cultural encounters; and 5) cultural desire. Cultural competence is conceived as a process that is acquired over time and with motivation. We have retained the model of Campinha-Bacote (2002) because of its relevance for the study of care processes where there are social class differences or a social distance.

We define social competence as a process based on knowledge, skills and attitudes that support effective interaction between the physician and patient despite the social distance that separates them. We take the five dimensions of Campinha-Bacote's cultural competence model and adapt them to the context of social difference: 1) social awareness; 2) social knowledge; 3) social skill; 4) social encounter; and 5) desire and motivation. Social awareness refers to the physician's consciousness of his class situation and of the prejudices and assumptions that exist about people living in poverty. Social knowledge includes acquiring, researching and obtaining information on social conditions, as well as on beliefs and values related to health. Social skill represents the capacity to adapt interventions in order to take into account the situations, needs and capacities of people on low incomes. Social encounter is the willingness to engage in a process of care with persons living in poverty. Finally, desire and motivation are affects (emotions) in the care process. They refer to the fact that the physician wants and is ready to engage in this care process in order to respond in a socially appropriate way to the needs of people coming from socially different circumstances.

**Figure 1:**
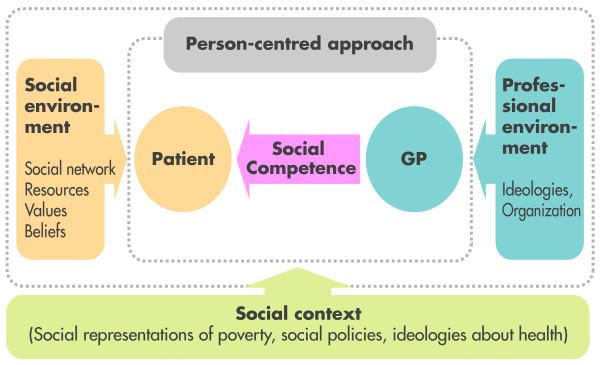
**Model of the social competence process (adapted from Campinha-Bacote, 2002; Stewart, 2003)**.

Figure 1 presents the model of the social competence process that we will use to guide our data collections. The diagram presents how social competence takes shape in the context of person-centred care. At the centre is social competence, which allows the physician to get closer to the patient. This competence makes it possible to narrow the social gap between them. This diagram includes the organizational and social environments and the larger social context that are part of the social factors that come into play in the provision of care. However, our study will be limited to examining those factors among physicians (i.e., professional ideologies, organizational context, social context).

### Research objectives

The aim of this project is to explore social competence among physicians providing primary care to people living in poverty. We want to understand the experience of physicians who provide care, on a daily basis, to people with low incomes who have at least one chronic illness. Our aim is to identify, from these physicians, the knowledge, skills and attitudes that support the social competence process.

## Methods/Design

This qualitative multiple-case study employs a sociological approach that allows for in-depth exploration of the perspectives of physicians who provide care daily to people living in poverty [37,39].

### Study population and sampling

The target population is made up of physicians providing care to low-income people in medical clinics located in disadvantaged neighbourhoods of the metropolitan Montreal region in Quebec (Canada). In other words, we want to focus on neighbourhoods where there is a high density of inhabitants on low incomes or living on welfare benefits.

Physicians will be recruited based on two criteria: they provide care to low-income patients with at least one chronic illness, and they have been identified by their peers as having developed expertise in providing care to a poor population. We will exclude physicians who offer mostly acute-care services or who work primarily in walk-in clinics.

One of the key sampling criteria in multiple-case studies that look at experiences and practices is the principle of *internal diversification *[40]. We wish to have, as key informants, physicians who devote their practice to low-income patients. These physicians will explain to us the evolution of their social competence process. Our aim is not to uncover everything that the physicians do in their practice with people living in poverty, but rather to describe in detail what the members of this subgroup of physicians who are accustomed to treating a low-income clientele have in common. Thus, we want to ensure that we have in our sample physicians of different ages, younger and older, both men and women, and who practise in a variety of organizational environments, in order to achieve intra-group diversity. By "organizational environment", we refer to the type of care-providing organization. This will allow us to achieve empirical-analytical generalizability, i.e., to be able to generalize the results from our subgroup to the entire population under study (physicians practising among low-income populations).

Based on our previous research among physicians and dentists, we estimate that 25 interviews are feasible and will be enough to meet the criteria for credibility (internal validity) and transferability (external validity). Nevertheless, we should point out that the size of the sample will be influenced by when we reach the saturation point (when new interviews no longer provide new information). Since data collection will be conducted iteratively and according to the needs of the analysis, the final number of participants may be slightly more or less than the 25 targeted.

### Data collection

We will carry out individual semi-structured interviews with each physician. The research assistant will interview every participant face-to-face. Interviews are expected to take 60 to 90 minutes and will be held in a location that is quiet and conducive to confidential discussion. The interview guide for the physicians will have three sections. The first section will cover the physician's professional career. The aim of this part of the interview is to create a climate of trust, and it is structured in such a way as to avoid social desirability bias. The second section will be devoted to the physician's experience with low-income patients. In this part of the interview, we begin to address the social encounter (SE), social skill (SS), and desire and motivation (DM) dimensions of social competence, drawn from the model adapted from Campinha-Bacote. The third section aims to explore the approach to care, and more specifically, how the physician approaches and provides care to a patient living in poverty. We will address the dimensions of social awareness (SA) and social knowledge (SK), and will go into more depth on the dimensions of social encounter (SE), social skill (SS), and desire and motivation (DM).

### Data analysis and interpretation

Data reduction [41] will be done using coding, which will consist of labelling, word by word, the different elements addressed in the interviews. We will use NVivo (QSR) software for the coding. We will prepare a summary list of codes for all the variables being studied. This list will be developed over the course of the analysis as new codes are inferred and pre-existing codes refined. The results will then be presented in tables summarizing the data obtained from each of the 25 interviews.

Our methodological approach will be characterized mainly by nearly simultaneous data collection and analysis, and by a recursive analytical process. Thus, the research assistant will code each interview as soon as the word-for-word transcription is finalized and will incorporate the data into analysis tables. The interpretations drawn from the tables and done by the research assistant and a researcher (Loignon) may feed back into the other phases of the analysis, entailing a refinement of the codes, if necessary, or a change in the interview plans, or, even further upstream, the selection of a particular profile. The data collection and analysis will continue in this manner until we reach the point of data saturation and stable conclusions.

We will ensure validity of the analyses and interpretations by employing triangulation procedures at every stage of the research. All members of the team will be invited to participate in the coding, following the first two interviews and toward the end of recruitment, in order to ensure saturation. Also, inter-coder reliability will be primarily ensured by the research assistant and a researcher (Loignon), both of whom will code every transcript independently and in parallel; they will then compare their results. Triangulation will also be applied to the production of hypotheses and the development of conclusions. To this end, the research agent and two researchers (Loignon and Allen) will meet four times to frame the interpretations. The whole team will meet twice during the analysis phase. Finally, it should be noted that all the data collection and analysis procedures will be described in research reports and will be open to rigorous methodological evaluation.

The validity of the analyses will also be strengthened through validation by the participants, which we will carry out by presentation of the results. This will be done by mailing each of the participants a summary report of our analysis results, accompanied by a letter advising them that the research agent will call them by telephone to obtain their opinion. We will then adjust or qualify our interpretations in accordance with the participants' feedback.

### Ethical considerations

This study is based on the usual ethical principles, such as every person's right to refuse to participate in the study and to withdraw at any time, as well as respect for all participants and protection of their privacy. Each person recruited will receive all the information necessary to provide free and informed consent. We will guarantee to the participants that our records will be kept in the strictest confidence, and we will ensure the confidentiality of all statements by assigning identification numbers to all participants to protect their identities. The records and audiotapes will be kept, sealed, at the *Hôpital Charles LeMoyne *(HCLM) research centre and will be destroyed at the end of five years. No names will appear on any public documents and we will take every precaution to divulge no information that would allow a third party to identify a participant. These ethical principles are clearly stated on the consent form, which was approved by the Committee for Ethics and Research of the *Centre hospitalier de l'Université Sherbrooke *(CHUS), Québec, Canada.

## Discussion

This study is one project (Project 1) in a research program aimed at improving the adequacy of health care for people living in poverty. This program includes two other research projects, one of which is funded by the FRSQ (2009); the three projects constitute a comprehensive whole. In effect, this first project will allow us to identify the dimensions of the social competence process that supports the creation of a therapeutic relationship between physicians and patients living in poverty with at least one chronic illness. However, these data from the interviews are the necessary groundwork for observation.

In fact, the identification of the dimensions of the social competence process will be very useful to frame our direct observations of the interactions that occur between physicians and their low-income patients. A second project (Project 2) will therefore involve in-clinic observations of the interactions between physicians (and other health professionals with whom they work) and low-income patients as well as interviews. Finally, a third project (Project 3) will consider the perspective of people living in poverty, their needs and expectations with regard to the approach of physicians and of the health care system.

This study will produce concrete recommendations for improving health interventions among low-income patients and for helping them to better manage their chronic illnesses. Ultimately, our aim is to strengthen the capacity of the health care system and of professionals to provide care that is adapted to the social conditions of people living in poverty. Residents and professors in family medicine will be informed of the results of our study. We will work in collaboration with our colleagues in the Department of Family Medicine at the *Université de Sherbrooke *(Québec, Canada) to enrich the teaching module on communication with patients by providing data on techniques or approaches that can improve the therapeutic alliance with patients living in poverty. An educational program to improve the social responsiveness of health care interventions will be developed and evaluated.

## Competing interests

The authors declare that they have no competing interests.

## Authors' contributions

CL conceived of the study, drafted the manuscript and coordinates the data collection and the analysis. JL, MF and CB participated in the design of the study and participate in the qualitative analysis. DA participates in the analysis of the data. DB participated in the design of the study and is involved in the collection of data. All authors read and approved the final manuscript.

## Pre-publication history

The pre-publication history for this paper can be accessed here:

http://www.biomedcentral.com/1472-6963/10/79/prepub

## References

[B1] Chalmers GW MacLeod KJ Thomson L Little SA McSharry C Thomson NC Smoking and airway inflammation in patients with mild asthma Chest 2001 120 1917 1922 10.1378/chest.120.6.1917 11742922

[B2] Chen Y Tang M Krewski D Dales R Relationship between asthma prevalence and income among Canadians JAMA 2001 286 919 920 10.1001/jama.286.8.919-a 11509055

[B3] Orpana HM Lemyre L Kelly S Do stressors explain the association between income and declines in self-rated health? A longitudinal analysis of the National Population Health Survey Int J Behav Med 2007 14 40 47 10.1007/BF02999226 17511532

[B4] Statistics Canada National Population Health Survey (NPHS) Asthma Supplement 1996/1997 Ottawa: Government of Canada

[B5] Ross NA Wolfson MC Dunn JR Berthelot JM Kaplan GA Lynch JW Relation between income inequality and mortality in Canada and in the United States: cross sectional assessment using census data and vital statistics BMJ 2000 320 898 902 10.1136/bmj.320.7239.898 10741994 PMC27328

[B6] Mercer SW Watt GC The inverse care law: clinical primary care encounters in deprived and affluent areas of Scotland Ann Fam Med 2007 5 503 510 10.1370/afm.778 18025487 PMC2094031

[B7] Lasser K Himmelstein DU Woolhandler S Access to care, health status and health disparities in the United States and Canada: results of a cross-national population-based survey American Journal of Public Health 2006 96 7 1300 1307 10.2105/AJPH.2004.059402 16735628 PMC1483879

[B8] Hutchison B Disparities in healthcare access and use: yackety - yack, yackety - yack Health Policy 2007 3 2 10 13 PMC2645179 19305775

[B9] Loignon C Représentations de la maladie, des traitements et conduites thérapeutiques: l'expérience de l'asthme [Doctoral Thesis] 2006 Montréal: Groupe de recherche interdisciplinaire en santé

[B10] Loignon C Bedos C Sévigny R Leduc N Understanding the self-care strategies of patients with asthma Patient Educ Couns 2009 75 255 262 10.1016/j.pec.2008.10.008 19041209

[B11] Mercer SW Cawston PG Bikker AP Quality in general practice consultations; a qualitative study of the views of patients living in an area of high socio-economic deprivation in Scotland BMC Fam Pract 2007 8 22 10.1186/1471-2296-8-22 17442123 PMC1857696

[B12] Reid C The Wounds of Exclusion: Poverty, Women's Health and Social Justice 2007 Walnut Creek, CA: Left Coast Press

[B13] Bedos C Brodeur JM Boucheron L Richard L Benigeri M Olivier M Haddas S The dental care pathway of welfare recipients in Quebec Soc Sci Med 2003 57 2089 2099 10.1016/S0277-9536(03)00066-2 14512240

[B14] Bedos C Brodeur JM Levine A Richard L Boucheron L Mereus W Perception of dental illness among persons receiving public assistance in Montreal Am J Public Health 2005 95 1340 1344 10.2105/AJPH.2004.045955 15985647 PMC1449364

[B15] Boltanski L Les usages sociaux du corps Les Annales 1971 1 205 233

[B16] Delruelle-Vosswinkel N Introduction à la sociologie générale, Brussels: Université de Bruxelles 1992

[B17] Hall JA Roter DL Katz NR Meta-analysis of correlates of provider behavior in medical encounters Med Care 1988 26 657 675 10.1097/00005650-198807000-00002 3292851

[B18] Milgrom P Hujoel P Grembowski D Fong R A community strategy for Medicaid child dental services Public Health Rep 1999 114 528 532 10.1093/phr/114.6.528 10670620 PMC1308536

[B19] O'Shea RM Corah NL Ayer WA Dentists' perceptions of the 'good' adult patient: an exploratory study J Am Dent Assoc 1983 106 813 816 6576016 10.14219/jada.archive.1983.0440

[B20] Rouse RA Hamilton MA Dentists evaluate their patients: an empirical investigation of preferences J Behav Med 1991 14 637 648 10.1007/BF00867176 1791626

[B21] Ventres W Gordon P Communication strategies in caring for the underserved J Health Care Poor Underserved 1990 1 305 314 2130910 10.1353/hpu.2010.0251

[B22] Malat J Expanding research on the racial disparity in medical tretament with ideas from sociology Health 2006 10 303 321 10.1177/1363459306064486 16775017

[B23] Parizot I Soigner les exclus. Paris: PUF 2003

[B24] Willems S De Maesschalck S Deveugele M Derese A De Maeseneer J Socio-economic status of the patient and doctor-patient communication: does it make a difference? Patient Educ Couns 2005 56 139 146 10.1016/j.pec.2004.02.011 15653242

[B25] Willems SJ Swinnen W De Maesseneer JM The GP's perceptions of poverty: a qualitative study Family Practice 2005 22 177 183 10.1093/fampra/cmh724 15710642

[B26] Price JH Desmond SM Snyder FF Kimmel SR Perceptions of family practice residents regarding health care and poor patients J Fam Pract 1988 27 615 621 3199090

[B27] Starr P The Social Transformation of American Medicine 1982 New York: Basic Books

[B28] Pineault R Lévesque J-F Roberge D Hamel M Lamarche P Haggerty J L'accessibilité et la continuité des services de santé: une étude sur la première ligne au Québec. Research Report. Québec: Institut National de santé publique du Québec 2008

[B29] Fiscella K Goodwin M Stange KC Does patient education level affect office visits to family physicians? J Natl Med Ass 2002 56 157 165 PMC2594098 11918385

[B30] Stewart M Reutter L Makwarimba E Rootman I Williamson D Raine K Wilson D Fast J Love R McFall S Shorten D Letourneau N Hayward K Masuda J Rutakumwa W Determinants of health-service use by low-income people Can J Nurs Res 2005 37 3 104 131 16268092

[B31] Ocean C Policies of Exclusion, Poverty and Health: Stories from the Front 2005 Duncan, BC: Wise Society

[B32] Cooper LA Roter DL Bone LR Larson SM Miller ER Barr MS Carson KA Levine DM A randomized controlled trial of interventions to enhance patient-physician partnership, patient adherence and high blood pressure control among ethnic minorities and poor persons: study protocol NCT00123045 Implement Sci 2009 19 4 7 10.1186/1748-5908-4-7 PMC2649892 19228414

[B33] Fernandez A Schillinger D Grumbach K Rosenthal A Stewart AL Wang F Pérez-Stable EJ Physician language ability and cultural competence: an exploratory study of communication with Spanish-speaking patients J Gen Intern Med 2004 19 2 167 174 10.1111/j.1525-1497.2004.30266.x 15009796 PMC1492135

[B34] Charlesworth SJ Gilfillan P Wilkinson R Living inferiority B Med Bull 2004 69 49-f 10.1093/bmb/ldh003 15226196

[B35] Allman RM Yoels WC Clair JM Clair JM, Allman RM Reconciling the agendas of physicians and patients Sociomedical Perspective on Patient Care 1993 Lexington, KY: University Press of Kentucky 29 46

[B36] Loignon C Allison PJ Landry A Richard L Brodeur JM Bedos C Providing socio-humanistic care: dentists' experience in deprived areas J Dent Res 2010 in press 10.1177/0022034510370822 20525962

[B37] Bennett M Paige RM Towards a developmental model of intercultural sensitivity Education for the Intercultural Experience 1993 Yarmouth, ME: International Press 109 135

[B38] Campinha-Bacote J The Process of Cultural Competence in the Delivery of Healthcare Services: a model of care J Transcult Nurs 2002 13 181 184 10.1177/10459602013003003 12113146

[B39] Willis J Foundations of Qualitative Research. Interpretive and Critical Approaches 2007 Thousand Oaks: Sage Publications

[B40] Pires A Poupart J, Groulx L-H, Deslauriers JP, Laperrière A, Mayer R Échantillonnage et recherche qualitative: essai théorique et méthodologique La recherche qualitative. Enjeux épistémologiques et méthodologiques 1997 Montréal: Gaëtan Morin 113 169

[B41] Miles MB Huberman AM Qualitative Data Analysis: An Expanded Sourcebook 1994 Thousand Oaks: Sage Publications

